# Relationship Between Inflammatory Markers (IL-6, Neutrophil–Lymphocyte Ratio, and C-Reactive Protein-Albumin Ratio) and Diabetic Ketoacidosis Severity: Correlation with Clinical Outcomes

**DOI:** 10.3390/medicina61020321

**Published:** 2025-02-12

**Authors:** Hatice Aslan Sirakaya, Hilal Sipahioglu, Ali Cetinkaya, Kaniye Aydin

**Affiliations:** 1 Department of Internal Medicine, The Kayseri City Hospital, Health Science University, Kayseri 38080, Turkey; dracetinkaya@gmail.com; 2 Department of Internal Medicine, Division of Medical Intensive Care Unit Kayseri, The Kayseri City Hospital, Health Science University, Kayseri 38080, Turkey; hilalgul1983@gmail.com; 3 Department of Internal Medicine, Division of Medical Intensive Care Unit, School of Medicine, Cukurova University, Adana 01330, Turkey; drkaniyeaydin@hotmail.com

**Keywords:** diabetic ketoacidosis, interleukin 6, CAR, NLR

## Abstract

*Background and Objectives*: The use of additional biomarkers to predict clinical course in diabetic ketoacidosis (DKA) is becoming increasingly important. The aim of this study was to investigate the relationship between interleukin-6 (IL-6) levels and the length of stay in the intensive care unit (ICU) in patients with DKA without signs of infection and to investigate the relationship between the neutrophil–lymphocyte ratio (NLR) and C-reactive protein (CRP) albumin ratio (CAR). *Materials and Methods*: This retrospective, single-center study included 78 patients with DKA without infection who were treated in the Medical ICU between July 2022 and June 2024. The patients were divided into two groups: moderate DKA (Group 1) and severe DKA (Group 2). The patients’ IL-6 levels, peripheral blood inflammatory markers (CAR, NLR), Acute Physiology and Chronic Health Evaluation (APACHE) II scores, and the duration of ICU stay were recorded. *Results*: The median duration of stay in the ICU was 2.00 (1–6) days in group 1 and 3.00 (1–26) days in group 2 (*p* = 0.001). The mean pH, HCO_3_, and CO_2_ values in Group 1 were 7.20 ± 0.07, 13.58 ± 2.11 mEq/L, and 29.45 ± 6.27 mmHg, while the mean pH, HCO_3_, and PCO_2_ values in Group 2 were 7.01 ± 0.11, 7.11 ± 1.91 mEq/L, and 20.35 ± 4.91 mmHg (*p* < 0.001, *p* < 0.001, *p* < 0.001, respectively). There was a strong positive correlation between IL-6 levels and the length of stay in the ICU (r = 0.813, *p* < 0.001). Additionally, there was a moderate positive correlation between the length of stay in the ICU with the severity of DKA (r = 0.475, *p* < 0.001), CAR (r = 0.336, *p* < 0.001), and NLR (r = 0.562, *p* < 0.001). *Conclusions*: Inflammatory markers such as NLR and CAR, and more specifically IL-6, were found to be associated with the clinical course and duration of stay in the ICU in patients with DKA.

## 1. Introduction

Diabetic ketoacidosis (DKA) is a serious metabolic complication that is also common in patients with type 2 diabetes mellitus (DM). While early diagnosis and intervention are critical in the management of DKA, the use of additional biomarkers to predict clinical outcome and optimize patient management is becoming increasingly important. Recent studies have reported a global increase in hospital admissions due to DKA in both type 1 and type 2 DM patients [1].

There are many studies suggesting that inflammation may play a role in the pathogenesis of DM [2,3,4]. In recent years, the role of inflammation in the pathophysiology of DKA has been emphasized [5,6]. Based on this, the effect of pro-inflammatory cytokines, especially interleukin-6 (IL-6) levels, on the severity and clinical outcomes of DKA has been investigated. IL-6 is a cytokine that stimulates the acute phase response and plays an important role in inflammatory processes [7,8]. It is known that increased IL-6 levels in patients monitored in the intensive care unit (ICU) are associated with poor prognosis [9,10]. However, there are limited data in the literature on the association of IL-6 levels with ICU length of stay and other inflammatory parameters in patients with DKA.

The evaluation of markers of inflammation such as neutrophil-to-lymphocyte ratio (NLR) and C-reactive protein (CRP)-to-albumin ratio (CAR) together with IL-6 may be potentially useful in determining the prognosis of patients with DKA. NLR is an indicator that reflects the acute inflammatory response and has been used to determine prognosis in various diseases [11,12]. Similarly, CAR is a parameter indicating the balance between inflammatory processes and nutritional status [13,14]. The aim of this study was to examine the relationship between IL-6 levels and length of ICU stay in patients with DKA without signs of infection and to investigate how this relationship is related to inflammatory markers NLR and CAR.

## 2. Materials and Methods

### 2.1. Study Design and Setting

This retrospectively designed, single-center study was conducted on 78 patients with DKA without infection hospitalized in the Medical ICU of Kayseri City Hospital, Kayseri, Türkiye. Approval for the study protocol was obtained from the local ethics committee with decision form number 224 and this study was conducted in accordance with the ethical principles outlined in the Declaration of Helsinki. Between July 2022 and June 2024, blood values of patients diagnosed with DKA in the Medical ICU were retrospectively analyzed.

### 2.2. Study Population

Patients with moderate and severe DKA (blood glucose level of 200 mg/dL and above, blood pH level below 7.25, serum bicarbonate level below 15 mEq/L, and urine-ketone-positive) who were hospitalized in the emergency department with a diagnosis of DKA; had no growth in blood, urine, or tissue cultures; had negative procalcitonin values; and where no source of infection was detected during physical examination were included in this study. Patients who developed DKA due to infection were not included in the study, while those who developed DKA due to treatment deficiency or treatment modification were included. Each patient was treated for DKA in accordance with the treatment protocol for adult patients with DKA [15].

**Figure 1:**
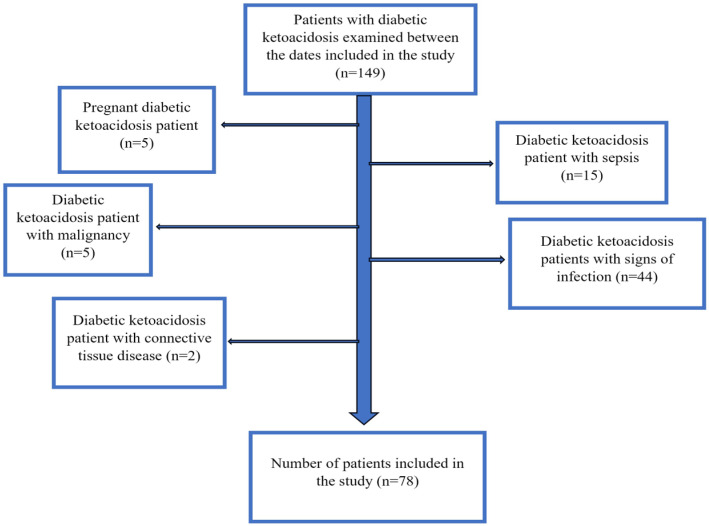
The flowchart of the patient selection process.

Patients with mild DKA and a hyperosmolar hyperglycemic state were excluded from this study. Patients under 18 years of age; pregnant patients; patients with connective tissue disease and malignancy; patients with positive growth in blood, urine, or tissue cultures, as well as those with elevated procalcitonin levels; patients with signs and foci of infection during physical examination; and patients diagnosed with sepsis according to sepsis-3 criteria were excluded [16] (Figure 1).

### 2.3. Data Collection and Outcome Measures

Patients’ clinical demographic data and laboratory results were recorded using the hospital information management system and archive files. Blood biochemistry, lipid panel and peripheral complete blood count, blood gas parameters, and IL-6 levels were recorded at the time of hospitalization. The IL-6 and other blood values of the patients were derived from the results of blood samples taken at the time of admission to the ICU. At the same time, the number of days of ICU stay, the duration of ICU stay and the total length of hospitalization, NLR and CAR rates, the anion gap, and plasma osmolarity were calculated. The absolute neutrophil count was divided by the absolute lymphocyte count to calculate the NLR. CAR values were obtained by dividing the CRP level by the serum albumin level. IL-6 level was determined using the ELISA (Thermo Fisher Scientific, Waltham, MA, USA) method. Patients were divided into two groups: moderate DKA (blood glucose level of 200 mg/dL and above, blood pH level below 7.0 to 7.25, serum bicarbonate level below 10 to <15 mEq/L) (Group 1) and severe DKA (blood glucose level of 200 mg/dL and above, blood pH level below < 7.0, serum bicarbonate level below <10 mEq/L) (Group 2). There were 32 patients in Group 1 and 46 patients in Group 2. Patients were grouped according to the 2024 Consensus report [14]. After the treatment of DKA, when the patients’ pH exceeded 7.3, bicarbonate levels rose above 18 mEq/L, and blood glucose dropped below 200 mg/dL, oral intake was initiated, and multiple-dose (basal-bolus) subcutaneous insulin therapy was started. Patients were then discharged from the ICU. The same discharge procedure was applied to all patients.

### 2.4. Statistical Analysis

All data analyses were performed using IBM SPSS (SPSS Inc., Chicago, IL, USA) version 22. The normal distribution of variables was evaluated using Kolmogorov–Smirnov tests and graphical analysis. When analyzing the study data, normally distributed variables are expressed as the mean and standard deviation, non-normally distributed variables are expressed as the median and interquartile range (IQR), and categorical variables are expressed as the percentage and number of cases. Student’s *t*-test was used to compare normally distributed variables between the two groups, and Kruskal–Wallis and Mann–Whitney U tests were used for variables that did not show normal distributions. Spearman’s correlation analysis was used to evaluate the univariate associations between the duration of ICU stay and IL-6 and inflammation parameters. To evaluate the effect of IL-6 on the length of ICU stay, a Poisson regression analysis was conducted using the Stata version 17.0 (StataCorp LLC, College Station, TX, USA) statistical software. The statistical significance level was accepted as *p* < 0.05 for all tests.

## 3. Results

A total of 78 patients, 62.8% (*n* = 49) of whom were female, were included in this study. There was no difference in gender distribution between the groups (*p* = 0.923). The mean age of the patients was 46.01 ± 19.82 years. The duration of ICU stay was 3.78 days. The median duration of ICU stay of the patients in the moderate-DKA group was 2.00 (1–6) days, the median duration of ICU stay of the patients in the severe-DKA group was 3.00 (1–26) days, and there was a significant difference between the groups (*p* = 0.001).The mean pH value of the patients in the moderate-DKA group was 7.20 ± 0.07, the mean pH value of the patients in the severe-DKA group was 7.01 ± 0.11, and there was a significant difference between the groups (*p* < 0.001). The mean HCO_3_ value of the patients in the moderate-DKA group was 13.58 ± 2.11 mEq/L, the mean HCO_3_ value of the patients in the severe-DKA group was 7.11 ± 1.91 mEq/L, and there was a significant difference between the groups (*p* < 0.001). The mean PCO_2_ value of the patients in the moderate-DKA group was 29.45 ± 6.27mmHg, the mean PCO_2_ value of the patients in the severe-DKA group was 20.35 ± 4.91 mmHg, and there was a significant difference between the groups (*p* < 0.001). IL-6 levels of the patients were 40.40 (5.04–460) pg/mL, IL-6 levels of the patients in the moderate-DKA group were 19.70 (5.60–73.10) pg/mL, IL-6 levels of the patients in the severe-DKA group were 50.35 (5.04–460) pg/mL, and there was a significant difference between the groups (*p* = 0.001) (Table 1).

**Figure 2:**
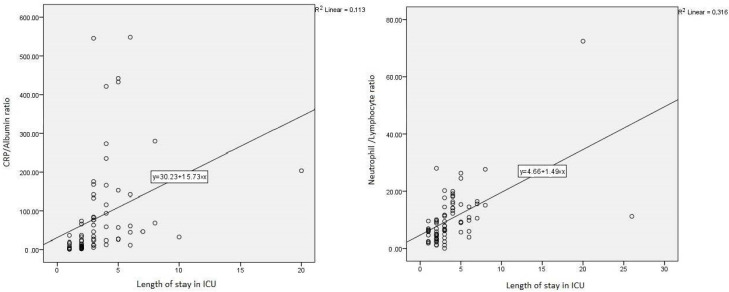
Correlation graph between length of stay in ICU with CAR and NLR.

A strong positive (*r* = 0.813) and significant correlation was found between IL-6 level and length of stay in the ICU (*p* < 0.001). A moderate positive correlation (*r* = 0.475, *r* = 0.336, *r* = 0.562, respectively) was found between the degree of DKA, CAR, NLR, and length of ICU stay (*p* < 0.001, *p* < 0.001, *p* < 0.001, respectively) (Table 2 and Figure 2).

Initially, to identify factors influencing the length of ICU stay, all variables were individually tested using Poisson regression analysis. Significant variables (*p* < 0.05), including IL-6, pH, and HCO_3_, were subsequently evaluated together to construct a regression model. To examine the linear relationship between pH and HCO_3_, a multicollinearity test was performed. The results of this test revealed that the VIF (Variance Inflation Factor) value for pH was 8.56, while for HCO_3_, it was 8.54. Due to high collinearity, pH was removed from the model. As a result, IL-6 and HCO_3_ were identified as independent risk factors for the length of ICU stay (*p* < 0.001 and *p* = 0.014, respectively) (Table 3).

## 4. Discussion

In this study, it was shown that there was a statistically significant relationship between IL-6 levels and length of ICU hospitalization in patients admitted to the ICU with a diagnosis of DKA. The finding that increased IL-6 levels were associated with longer ICU stay in DKA patients suggests that IL-6 may be an indicator of the severity of DKA. Our study suggests that IL-6 plays a determinant role not only in the acute phase response but also in the clinical course of severe metabolic disorders such as DKA. Another noteworthy finding of our study was the significant correlation between IL-6 levels and other inflammatory markers such as NLR and CAR. Additionally, IL-6 has been shown to be an independent risk factor for ICU length of stay.

In the literature, IL-6 has been reported to be effective on prognosis in infectious conditions as well as in various acute metabolic and stress conditions in which inflammatory burden increases [17]. Therefore, evaluating the effect of inflammatory processes on prognosis in DKA with IL-6 levels may provide important clues in the management of the disease. In conditions characterized by high metabolic stress and hyperglycemia such as DKA, investigating the effects of inflammatory markers such as IL-6 on the duration of ICU stay and the general recovery process may contribute to the development of strategies to improve clinical outcomes [7]. In this study, the fact that IL-6 was associated with the duration of ICU stay in DKA patients suggests that these patients can be managed more predictably if IL-6 levels are included in the clinical evaluation process. In recent years, some supplements have been shown to reduce hyperglycemia in the literature [18]. Although the exact mechanism of this effect is not fully understood, it is thought to be related to theiranti-inflammatory properties. Similarly, the use of certain supportive supplements may be considered in conditions where inflammation is increased, such as DKA.

The literature consistently indicates that elevated IL-6 levels are associated with poor prognostic outcomes in various critical illnesses [19,20,21]. Similarly, the central role of IL-6 in inflammatory processes in DKA patients goes in parallel with the worsening clinical picture of these patients. While IL-6 regulates the immune response triggered by pro-inflammatory cytokines, it can also be considered an indicator of metabolic stress, insulin resistance, and endothelial dysfunction. Previous studies have shown that IL-6 levels are associated with insulin resistance and hyperglycemic states, especially in diabetic individuals [8]. Therefore, increased IL-6 may contribute to the severity of the disease as a combination of both inflammation and metabolic disorders.

Previous studies have investigated the elevation and significance of IL-6 in patients with infection and sepsis-related DKA [7,22]. In these patients, CRP and IL-6 were found to increase due to sepsis, and its effect on prognosis was emphasized. In the present study, we have shown that IL-6 is an independent inflammatory marker because we evaluated DKA patients excluding sepsis and infection.

Elevated IL-6 levels reflect the activation of the inflammatory cascade, which could exacerbate metabolic dysregulation and contribute to delayed recovery. While inflammation in DKA is often viewed as a secondary response to metabolic stress, our findings suggest that it may actively influence the severity of the condition. This hypothesis aligns with prior evidence highlighting the role of inflammatory cytokines in acute metabolic decompensation [8].

In the literature, various inflammatory markers have been used to assess the severity of many diseases. Furthermore, studies on the roles of inflammatory markers have explored age-related changes in certain inflammatory parameters in healthy individuals [23]. Inflammatory parameters have been investigated in a wide range of diseases, from DM to embolism [3,24]. One of these inflammatory parameters, NLR, is a commonly used parameter as an indicator of systemic inflammation and immune response [11,12,25]. It is known that increased NLR is associated with poor clinical outcomes in various critical illnesses. In our study, the positive correlation between IL-6 levels and NLR suggests that the clinical course may worsen in patients with a stronger inflammatory response. Increased NLR is an indicator of immune cell imbalance and inflammatory burden, and may be among the factors leading to longer ICU stays in these patients. In addition, CAR is another important marker reflecting the relationship between inflammatory processes and nutritional status [14]. While increased CRP indicates the severity of the inflammatory response, low albumin levels are generally considered a sign of malnutrition and catabolic processes. High CAR indicates that inflammation is high and the nutritional status is poor in DKA patients. The correlation between IL-6 and CAR shows that nutritional status may deteriorate further as inflammation becomes more severe, and this situation may increase the severity of the disease and prolong the duration of ICU stay. In this context, the combined evaluation of inflammatory markers may provide more sensitive results in terms of patient management and prognosis.

The findings of our study suggest that inflammatory markers such as IL-6, NLR, and CAR may be important in predicting clinical outcomes of patients with DKA and optimizing ICU management. The use of these biomarkers may contribute to the development of individualized approaches in the treatment of patients with DKA. In particular, implementing more aggressive treatment strategies or introducing more intensive monitoring protocols in patients with elevated IL-6 levels may improve clinical outcomes.

Our study has some limitations. First of all, the retrospective design and single-center study may limit the generalizability of the results. In addition, further prospective and multicenter studies are required to further examine the effects of IL-6 on the clinical course. Nevertheless, our study provides an important perspective on the role of IL-6 in DKA and emphasizes the importance of including inflammatory processes in clinical decisionmaking in these patients.

## 5. Conclusions

In conclusion, it was observed that inflammatory markers such as IL-6, NLR, and CAR in patients with DKA were associated with clinical course and ICU length of stay. It was also observed that IL-6 had a more pronounced relationship with these results than the others. A validation of these biomarkers in larger studies may contribute to the management of DKA. In the future, such inflammatory parameters may serve as potential markers for evaluating the clinical course.

## Figures and Tables

**Table 1:** Comparison of demographic and clinical findings of the groups.

	All Patients (*n*:78)	Moderate DKA (*n*:32)	Severe DKA (*n*:46)	*p*-Value
Sex (male/female)	29/49	11/21 (34.4/65.6)	18/28 (39.1/60.9)	0.923
Age (years)	46.01 ± 19.82	44.56 ± 20.58	45.02 ± 19.55	0.673
Intensive Care Hospitalization Duration (days)	3.78 (1–26)	2.00 (1–6)	3.00 (1–26)	0.001
GCS score	13.92 ± 1.06	14.03 ± 1.06	13.84 ± 1.07	0.458
APACHE II score	22.85 ± 5.83	21.78 ± 6.00	23.60 ± 5.65	0.175
NUTRIC score	3.78 ± 1.69	3.56 ± 1.72	3.93 ± 1.67	0.109
BMI (kg/m^2^)	24.12 ± 2.28	24.42 ± 2.25	23.84 ± 2.33	0.889
Glukoz (mg/dL)	573.55 ± 198.29	567.89 ± 236.228	596.76 ± 182.63	0.698
HbA1C (%)	11.68 ± 2.43	11.88 ± 2.51	11.88 ± 2.25	0.995
CRP (mg/L)	17.05 (0.3–386)	8.90 (0.6–386)	19.05 (0.3–292.5)	0.541
Albumin (mg/dL)	38.50 ± 7.67	37.19 ± 7.89	38.93 ± 7.54	0.656
pH	7.09 ± 0.13	7.20 ± 0.07	7.01 ± 0.11	<0.001
HCO_3_ (mEq/L)	9.71 ± 3.70	13.58 ± 2.11	7.11 ± 1.91	<0.001
PCO_2_ (mmHg)	24.46 ± 7.09	29.45 ± 6.27	20.35 ± 4.91	<0.001
Lactate (mmol/L)	3.05 (1.20–12.10)	2.80 (1.20–12.10)	3.25 (1.20–9.40)	0.615
White blood cell (10^3^/μL)	15.89 ± 8.90	14.49 ± 7.82	17.56 ± 9.84	0.995
Absolute neutrophil count (10^3^/μL)	13.37 ± 7.96	11.77 ± 7.26	15.10 ± 8.55	0.106
Absolute lymphocyte count (10^3^/μL)	1.76 ± 1.02	1.75 ± 1.17	1.81 ± 0.94	0.941
CRP/albumin ratio	35.82 (0.57–1289.67)	23.12 (1.94–1289.67)	51.00 (0.57–1063.33)	0.600
Neutrophil/lymphocyte ratio	8.89 (0.03–72.43)	9.28 (1.11–72.43)	9.03 (0.03–27.64)	0.473
Interleukin 6 (pg/mL)	40.40 (5.04–460)	19.70 (5.60–73.10)	50.35 (5.04–460)	0.001

GCS: Glasgow coma scale, APACHE II: Acute Physiology and Chronic Health Evaluation II, NUTRIC score: Nutrition Risk in Critically ill score, BMI: body mass index, CRP: C-reactive protein. Values are presented as number (%) of patients, mean ± standard deviation, or median (minimum–maximum).

**Table 2:** Results of the correlation analysis of factors associated with the length of stay in the intensive care unit.

	r-Value	*p*-Value
IL-6	0.813	<0.001
Degree of ketoacidosis	0.475	<0.001
CRP/albumin ratio	0.336	<0.001
Neutrophil/lymphocyte ratio	0.562	<0.001

Correlation is significant at the *p* < 0.05 level.

**Table 3:** Evaluation of independent risk factors affecting ICU length of stay using Poisson regression analysis.

	IRR	z	*p*	95% CI
IL-6	1.04	7.77	<0.001	1.02–1.06
HCO_3_	−0.44	−2.47	0.014	−0.80–−0.93

IRR: Internal Rate of Return, CI: confidence interval.

## Data Availability

The datasets generated and/or analyzed during the current study are not publicly available, as they contain patient information, but the data that support the findings of this study are available from the corresponding author (HAS) on reasonable request.

## Abbreviations

The following abbreviations are used in this manuscript:

DKA	Diabetic ketoacidosis
DM	Diabetes mellitus
ICU	Intensive care unit
NLR	Neutrophil-to-lymphocyte ratio
CAR	CRP-to-albumin ratio
IQR	Interquartile range
VIF	Variance Inflation Factor
